# Responses of hepatic sinusoidal cells to liver ischemia–reperfusion injury

**DOI:** 10.3389/fcell.2023.1171317

**Published:** 2023-04-04

**Authors:** Yoshiya Ito, Kanako Hosono, Hideki Amano

**Affiliations:** Department of Pharmacology, Kitasato University School of Medicine, Kanagawa, Japan

**Keywords:** liver repair, ischemia-reperfusion, macrophage, endothelial cells, stellate cell

## Abstract

The liver displays a remarkable regenerative capacity in response to acute liver injury. In addition to the proliferation of hepatocytes during liver regeneration, non-parenchymal cells, including liver macrophages, liver sinusoidal endothelial cells (LSECs), and hepatic stellate cells (HSCs) play critical roles in liver repair and regeneration. Liver ischemia–reperfusion injury (IRI) is a major cause of increased liver damage during liver resection, transplantation, and trauma. Impaired liver repair increases postoperative morbidity and mortality of patients who underwent liver surgery. Successful liver repair and regeneration after liver IRI requires coordinated interplay and synergic actions between hepatic resident cells and recruited cell components. However, the underlying mechanisms of liver repair after liver IRI are not well understood. Recent technological advances have revealed the heterogeneity of each liver cell component in the steady state and diseased livers. In this review, we describe the progress in the biology of liver non-parenchymal cells obtained from novel technological advances. We address the functional role of each cell component in response to liver IRI and the interactions between diverse immune repertoires and non-hematopoietic cell populations during the course of liver repair after liver IRI. We also discuss how these findings can help in the design of novel therapeutic approaches. Growing insights into the cellular interactions during liver IRI would enhance the pathology of liver IRI understanding comprehensively and further develop the strategies for improvement of liver repair.

## Introduction

Liver ischemia–reperfusion injury (IRI) is a major cause of liver damage during liver resection, transplantation, and trauma. Although acute liver injury initiates a regenerative response, acute liver failure is induced when liver IRI impairs the process of liver repair and regeneration. Hence, the regenerative ability of the remnant liver determines the outcomes of liver surgery. Growing evidence suggests that inadequate liver repair and regeneration culminate in postoperative liver dysfunction, which is associated with poor patient outcomes (Lentsch, 2012; Bagante et al., 2019). Aggravation of liver IRI can affect liver transplant outcomes by increasing the incidence of primary graft dysfunction and both acute and chronic rejection (Zhai et al., 2013). Therapeutic strategies that stimulate liver repair and prevent liver IRI would enhance the regenerative capacity of the liver. The molecular mechanisms of the inflammatory responses to liver IRI has been extensively investigated, contributing to a better understanding of the disease (Hirao et al., 2022); however, the resolution and outcomes of liver regeneration after liver IRI remain largely unknown.

The liver is comprised of parenchymal and non-parenchymal cells. Parenchymal cells account for approximately 60% of the total cell population in the human liver, with non-parenchymal cells constituting the rest of the cells. Major components of liver non-parenchymal cells include resident macrophages (Kupffer cells, KCs) and liver sinusoidal endothelial cells (LSECs), and hepatic stellate cells (HSC, liver specific pericytes), all of which play important roles in maintaining homeostasis (van Golen et al., 2012; Krenkel and Tacke, 2017). The development of liver IRI affects all resident sinusoidal cell populations, especially macrophages, LSECs, and HSCs. Liver repair and regeneration after liver IRI are characterized by the removal of damaged tissue and the recovery of the function and structure of non-parenchymal cells. Each cellular component in the liver participates in the process of liver repair after IRI. Among these cells, macrophages play a crucial role in stimulating liver repair and regeneration after liver IRI (Hirao et al., 2022). Prevention of macrophage accumulation in the injured regions with blockade of vascular endothelial growth factor receptor 1 (VEGFR1) or leukotriene receptor 1 impairs hepatocyte proliferation and delays liver repair in mice (Ohkubo et al., 2013; Ohkubo et al., 2014). It was also reported that macrophage plasticity is essential for liver repair after acute liver injury induced by IRI (Nishizawa et al., 2018), N-acetyl-para-aminophenol (APAP) (Holt et al., 2008; Zigmond et al., 2014), and sterile heat (Dal-Secco et al., 2015).

Advances in single-cell and spatial transcriptomic technologies have broadened the understanding of functional heterogeneity not only of macrophages (Guilliams and Scott, 2022) but also of LSECs (Halpern et al., 2018) and HSCs (Dobie et al., 2019) in steady states and diseased conditions (Ramachandran et al., 2019; Krenkel et al., 2020). A variety of signaling networks are active between non-parenchymal cells during liver IRI. Understanding the contributions of different cell types in the process of liver repair will provide new perspectives toward the pathology of IRI and the development of therapeutic strategies that target specific cell components for effective liver repair following IRI.

In this review, we summarize the current knowledge of the heterogeneity of hepatic macrophages, LSECs, and HSCs during homeostasis and their functional roles in liver repair after IRI through interactions with other immune cells. We will discuss the potential implications for promoting liver regeneration from liver IRI. A better understanding of these cellular mechanisms will deepen our understanding of the pathology of liver repair after IRI.

## Macrophages in liver repair

**FIGURE 1:**
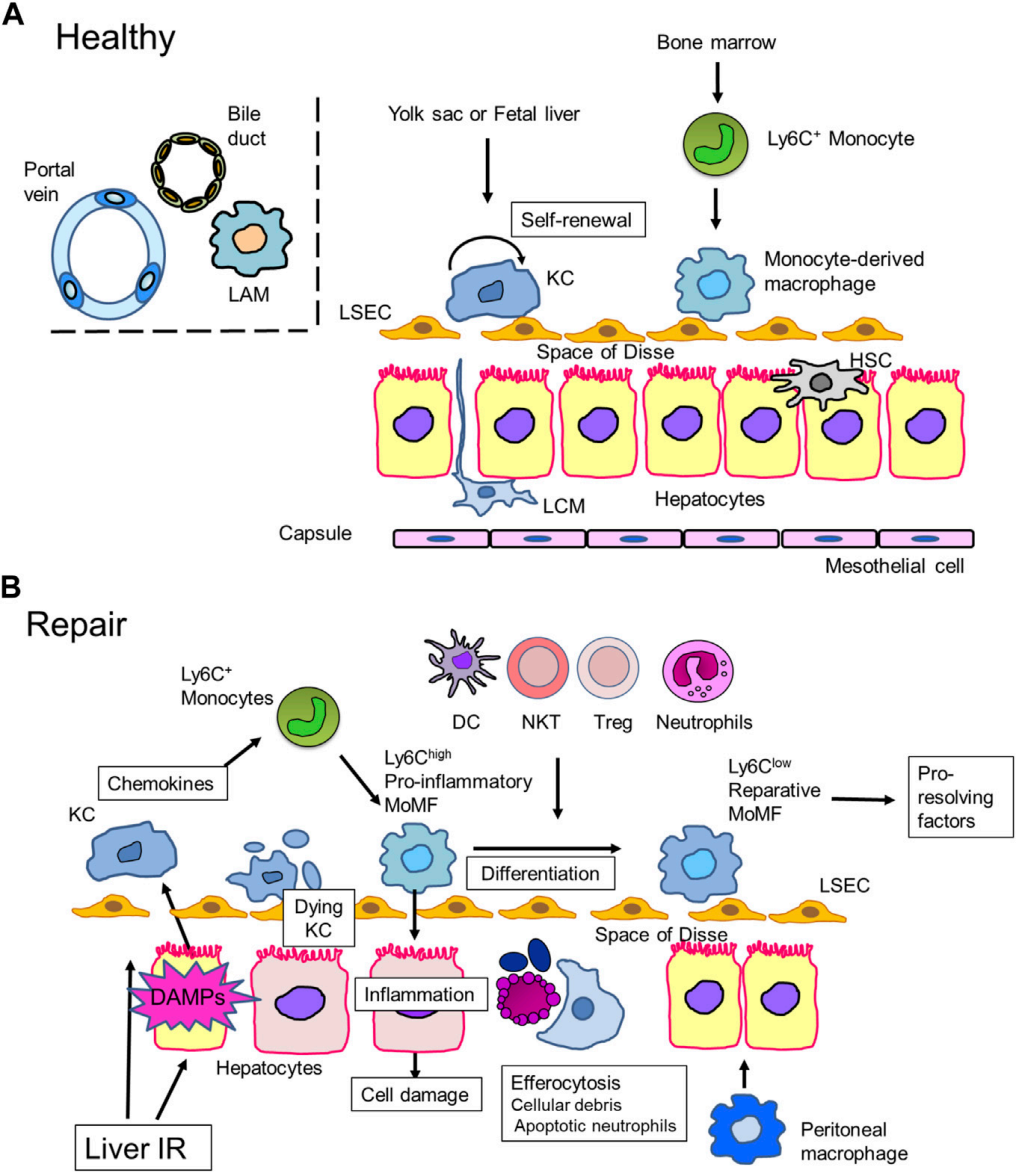
Macrophages in liver repair **(A)**. Liver macrophages in steady state. In healthy mice livers, most liver macrophages consist of self-renewing resident macrophages, Kupffer cells (KCs), which reside in liver sinusoids. KCs are derived from progenitor cells in the yolk sac or fetal liver. In addition to KCs, mouse livers contain a small proportion of recruited monocyte-derived macrophages, which arise from bone marrow-derived Ly6C^+^ monocytes. Other discrete populations of macrophages can be found at the liver capsule, called liver capsular macrophages (LCMs), and at the bile-duct, called (bile-duct) lipid-associated macrophages (LAMs). LCMs protect the liver from peritoneal pathogens, while the role of bile-duct LAMs remains elusive. In contrast, human livers consist of both KCs and recruited monocyte-derived macrophages. **(B)**. Liver IRI-induced hepatocyte damage leads to the release of inflammatory mediators, including danger-associated molecular patterns (DAMPs), which activate Kupffer cells (KCs) to secrete chemokines, including CCL2. Damaged KCs undergo cell death through necroptosis, pyroptosis, or ferroptosis. Ly6C^high^ monocytes are mobilized from the bone marrow and recruited to the site of injury. Monocytes then differentiate into monocyte-derived macrophages (MoMFs), which exhibit a pro-inflammatory phenotype (pro-inflammatory MoMF). Pro-inflammatory MoMFs replenish the KC population and initiate inflammation to cause hepato-cellular damage. Pro-inflammatory MoMFs also help remove dead hepatocytes, KCs, and apoptotic neutrophils. *In situ* macrophage phenotype switching from a pro-inflammatory to a reparative phenotype is facilitated by efferocytosis and crosstalk with other immune cells. Reparative macrophages contribute to liver repair and regeneration by producing pro-resolving factors. Peritoneal macrophages cross the mesothelium and accumulate at the site of liver injury to support liver repair.

Liver macrophages play a crucial role in the maintenance of liver homeostasis and liver inflammation and resolution. Accumulating evidence indicates that liver macrophages display tremendous heterogeneity with distinct functions and gene signatures in normal and diseased livers. Here, we review the roles of liver macrophages in liver repair and regeneration after liver IRI (Figure 1).

### Macrophages in healthy livers

Hepatic macrophages are predominantly composed of liver-resident macrophages, traditionally known as KCs, which play a central role in maintaining homeostasis. KCs are the most abundant tissue macrophages in mammalian bodies, and 80%–90% of all macrophages in the human body reside in the liver (van Golen et al., 2012). In the liver, KCs constitute approximately 15% of the liver cells and are distributed along the hepatic sinusoids especially near peri-portal areas. This distribution appears to be directed by LSECs sensing the presence of microbial products (Gola et al., 2021). Recent studies have revealed that KCs that reside in the hepatic sinusoids extend a portion of their body into the space of Disse and closely come in contact with LSECs, HSCs, and hepatocytes (Bonnardel et al., 2019). Given their anatomical position, KCs constitute one of the first lines of defense against pathogens. KCs express complement receptors of the immunoglobulin superfamily (CRIg) and rapidly recognize and phagocytose bacteria from the portal veins (Helmy et al., 2006). CRIg is predominantly and highly expressed in the liver. Accordingly, KCs are responsible for eliminating damaged cells through phagocytosis, prevent systemic and intestinal-derived pathogens, and regulate iron metabolism. KCs also act as antigen-presenting cells and regulate adaptive immune responses. To maintain an anti-inflammatory microenvironment, KCs induce anti-inflammatory cytokine production through their interaction with activated regulatory T cells (Tregs) and suppression of effector T cell activation, resulting in hepatic immune tolerance. Recent evidence suggests that KCs are mainly derived from the yolk sac or the fetal liver (Yona et al., 2013; Gomez Perdiguero et al., 2015; Hoeffel et al., 2015). KCs are characterized by the expression of F4/80, CD11b^low^, C-type lectin domain family 4 member F (CLEC4F), and V-set and immunoglobulin domain-containing 4 (VSIG4) in mice (Scott et al., 2016; Guilliams et al., 2022). Additionally, murine KC-specific markers include CLEC4F, VSIG4, CLEC2, and FOLR2 (Guilliams et al., 2022). Under steady state conditions, the local population of KCs is maintained by self-renewal (McDonald and Kubes, 2016) independent of bone marrow (BM)-derived progenitors (Yona et al., 2013; Hoeffel et al., 2015). In the human liver, single-cell RNA-sequencing studies identified two distinct KC subsets (MacParland et al., 2018; Ramachandran et al., 2019). For instance, CD68^+^ KCs consist of macrophage receptor with collagenous structure-expressing (MARCO^+^) and MARCO^−^. CD68^+^MARCO^+^ KCs were enriched in genes related to immunoregulation (VSIG4, CD163, and HMOX1 (hemoxygenase) and CD68^+^MARCO^−^ KCs were enriched in genes related to pro-inflammation (S100A8/9, IL-18, and C1QC), suggesting functional zonation of macrophage subsets (MacParland et al., 2018). The former appears to be similar to self-renewal KCs identified in mice, while the latter is likely monocyte-derived macrophages (MacParland et al., 2018). VSIG4 is expressed in humans as well as mice.

In addition to KCs, BM-derived monocytes expressing lymphocyte antigen 6 complex locus C1 (Ly6C) migrate from the bloodstream into liver tissues, giving rise to monocyte-derived macrophages (MoMFs) (Wen et al., 2021). In contrast to KCs, a small population of MoMFs is located mainly in the portal triad (Gammella et al., 2014; Theurl et al., 2016). However, circulating monocytes through the hepatic sinusoids barely contribute to the adult KC pool under steady state conditions (Schulz et al., 2012; Yona et al., 2013; Scott et al., 2016). When the KC pool is depleted, MoMFs contribute to maintaining the KC pool (Schulz et al., 2012). MoMFs in mice are largely found in inflamed livers (David et al., 2016; Daemen et al., 2021) or in livers under experimental KC depletion by administration of clodronate liposomes (Soysa et al., 2019) and diphtheria toxin (DT) (Scott et al., 2016). Circulatory monocytes, which are derived from BM-resident hematopoietic stem cells (Hashimoto et al., 2013; Eaves, 2015), are recruited to replace KCs (Bleriot et al., 2015). The recruited MoMFs acquire a phenotype similar to that of KCs when an empty niche is available (Beattie et al., 2016; Scott et al., 2016; Bleriot and Ginhoux, 2019). The recruitment of monocytes from BMs is induced by toll-like receptors (TLR) in KCs or HSCs, resulting in the production of chemokine (C-C motif) ligand 2 (CCL2) (Mossanen et al., 2016). Meanwhile, in the healthy adult human liver, the number of MoMFs is greater than that in healthy mouse liver (Guilliams et al., 2022). The exposure to numerous stimuli, including gut-derived pathogens, drugs, and alcohol, may result in MoMFs settling in the liver and maintaining a pool of resident macrophages.

Recently, another population of resident liver macrophages residing in the hepatic capsule, namely, liver capsular macrophages (LCMs), has been identified in mice (Sierro et al., 2017). LCMs are phenotypically and developmentally different from KCs and MoMFs. In the murine liver, LCMs are not derived from embryonic precursors and do not self-renew. Instead, LCMs are derived from circulating monocytes and are replenished. LCMs express general markers for macrophages (F4/80 and CD11 b) but not for KCs (Clec4F and T-cell immunoglobulin and mucin domain containing 4 (Tim 4) (Sierro et al., 2017; Guilliams et al., 2022). It is suggested that LCMs are the first line of defense against peritoneal pathogens. LCMs recognize peritoneal bacteria by assessing the liver capsule and facilitating neutrophil recruitment. Ablation of LCMs reduces neutrophil recruitment and increases the number of intrahepatic pathogens from the peritoneal cavity. However, LCMs have not been identified in the human liver.

Recent studies have demonstrated the presence of other populations of liver macrophages around the bile ducts in both mice and healthy human livers (Guilliams et al., 2022). These macrophages express genes related to lipid metabolism (glycoprotein nmb *(Gpnmb*) and *Spp1*), which are also expressed in macrophages in mouse livers with non-alcoholic steatohepatitis (NASH) and obese adipose tissue, and are termed lipid-associated macrophages (LAMs) (Remmerie et al., 2020). LAMs located around the bile ducts are referred to bile-duct LAMs, which are induced by local lipid exposure (Guilliams et al., 2022) (Figure 1A).

### KCs in liver IRI

Liver IRI induces cellular damage, resulting in the release of endogenous molecules such as danger-associated molecular patterns (DAMPs). Several nuclear, cytosolic, and mitochondrial molecules have been identified as DAMPs (Hirao et al., 2022). DAMPs released from dead or dying cells at the site of injury activate immune cells through pattern-recognition receptors (PRRs), including TLRs and nucleotide binding oligomerization domain-like receptors. In the early phase of liver IRI, DAMPs from injured hepatocytes activate KCs through PRRs to produce inflammatory mediators, such as chemokines, cytokines, and reactive oxygen species (ROS) (Jaeschke, 2003), leading to the initiation and progression of liver IRI (Shan and Ju, 2020; Hirao et al., 2022). For example, damaged hepatocytes secrete high mobility group box 1 (HMGB1) that binds to TLRs to activate KCs to mediate liver IRI (Tsung et al., 2005; Bamboat et al., 2010). Anti-HMGB1 neutralizing antibodies or thrombomodulin (an HMGB1 inhibitor) mitigates hepatocellular damage and modulates the effects of pro-inflammatory mediators in a mouse liver IRI model (Tsung et al., 2005; Kadono et al., 2017). In addition to HMGB1, other DAMPs, including histones, DNA fragments, adenosine triphosphate, and mitochondrial ROS, also activate KCs *via* different PRRs to produce pro-inflammatory mediators that exacerbate IRI (Shan and Ju, 2020). Furthermore, inhibition of PRRs using TLR antagonists attenuated liver inflammation. Notably, pre-treatment with a TLR4 antagonist ameliorated liver IRI in murine models (McDonald et al., 2015).

To understand whether KCs mediate inflammation and/or resolution in response to liver IRI, livers with pharmacological depletion of KCs have been evaluated. Pre-treatment with gadolinium chloride attenuated liver IRI in rats (Hisama et al., 1996). In contrast, administration of clodronate liposomes to deplete KCs at 24 h (Devey et al., 2009) and 48 h (Yue et al., 2017) before liver ischemia aggravated liver IRI in mice. Additionally, KC with inhibition of TIM-4 at 48 h before liver ischemia exacerbated liver IRI in mice, while TIM-4 inhibition at 2 h before induction of ischemia attenuated liver IRI (Ni et al., 2021). Pre-treatment with clodronate liposomes or anti-TIM-4 antibodies 24 h or 48 h prior to injury induction not only depletes KCs but also induces the accumulation of pro-inflammatory macrophages in the liver because of KC depletion. The recruitment of pro-inflammatory macrophages into the liver would then exacerbate liver IRI. A similar finding was reported in acute liver injury induced by APAP administration in mice (Shan et al., 2021), demonstrating that pre-treatment with clodronate liposomes exacerbated APAP-induced liver injury due to the recruitment of pro-inflammatory macrophages at the time of APAP administration. Thus, these experimental approaches with clodronate liposomes or neutralizing antibodies might recruit other types of immune cells, including macrophages and neutrophils, which are not present in steady state murine livers. These findings suggest that acute liver injury recruits pro-inflammatory MoMFs and aggravate liver inflammation. Off-target effects of KC deletion on immune cell characterization should be considered for potential drawbacks and for the interpretation of experimental data (Zwicker et al., 2021). It is critical to understand whether KC loss is essential for the regulation of liver inflammation or is a consequence of inflammation.

### Mode of KC death after liver IRI

The induction of liver IRI reduces the number and proportion of resident KCs (Ohkubo et al., 2014; Yue et al., 2017; Nishizawa et al., 2018; Goto et al., 2021). The reduction or loss of KCs is a common feature of liver diseases, including APAP-induced liver injury (Kato et al., 2011; Zigmond et al., 2014; Ben-Moshe et al., 2022) and bacterial or viral infections (Bleriot et al., 2015; Borst et al., 2018).

During the early phase of liver IRI, KCs undergo cell death. As dying cells release pro-inflammatory or pro-resolution-of-inflammation mediators, KC death plays a critical role in orchestrating the progression and/or resolution of inflammation during liver IRI. As macrophage death regulates liver inflammation (Li and Weinman, 2018), understanding the mode of cell death is important. In addition to apoptosis and necrosis, there are several modes of programmed cell death, including necroptosis (Linkermann and Green, 2014), pyroptosis (Shi et al., 2017), and ferroptosis (Dixon et al., 2012), have suggested involvement in liver IRI (Hirao et al., 2022) (Figure 1B). However, the contribution of any mode of KC death to the mechanisms of liver IRI and their role in liver resolution and repair remains controversial (Wen et al., 2021).

The contribution of apoptotic cell death in caused by liver IRI has been controversial. Apoptosis, as determined by the increased expression of terminal deoxynucleotidyl transferase dUTP nick end labeling (TUNEL) and caspase activities, play an important role in liver IRI (Ke et al., 2013); however, caspase-3 activation has not been demonstrated during liver IRI, and an apoptotic marker (caspase-cleaved fragment of cytokeratin 18) is lacking (Yang et al., 2014). Because DNA fragmentation leads to secondary necrosis, TUNEL is observed in hepatic cell death in both apoptosis and necrosis (Jaeschke and Lemasters, 2003). Based on the findings that dying hepatocytes display morphological features characteristic of necrosis, the mode of cell death during liver IRI appears to be necrotic and not apoptotic. Thus, liver IRI-induced KC death is likely due to necrosis.

Necroptosis is a specific form of programmed necrosis that is mediated by receptor-interacting serine/threonine-protein kinase (RIPK)-1, RIPK-3, and the downstream molecule mixed-lineage kinase domain-like protein (Linkermann and Green, 2014). RIPK-3-deficient mice exhibited amelioration of liver IRI through reduced neutrophil accumulation (Ni et al., 2019). During the early phase of liver IRI, RIPK-1 and RIPK-3 levels in KCs (Yue et al., 2017) and RIPK-3 levels in the liver were increased (Hong et al., 2016). Additionally, the RIPK-1 inhibitor necrostatin-1 prevented KC death and mitigated liver IRI; however, other reports failed to demonstrate these findings (Saeed et al., 2017).

Ferroptosis, a newly discovered form of cell death characterized by iron-dependent lipid peroxidation (Dixon et al., 2012), was suggested to be involved in liver IRI-induced cell death (Yamada et al., 2020). Although ferroptosis appears to contribute to the progression of liver IRI, it remains unclear whether ferroptosis in KCs or macrophages is responsible for liver IRI.

Recently, pyroptosis, a mechanism of programmed cell death that depends on gasdermin processing (Shi et al., 2017), was suggested to be a relevant cell death pathway in liver IRI (Kadono et al., 2022). Increased levels of gasdermin D (GSDMD) and caspase-1, a critical mediator for pyroptosis, in recruited macrophages aggravated liver IRI *via* activation of the inflammasome-mediated pyrolysis pathway as indicated by the increased production of IL-1β and IL-18. Consistent with this finding, caspase-1 inhibitor and myeloid cell-specific GSDMD deletion mitigated liver IRI (Li et al., 2020). However, GSDMD cleavage and IL-1β/IL-18 release may not always occur following macrophage death (Evavold et al., 2018). Pyroptosis may be a mode of cell death relevant for liver IRI; however, further investigation is needed to determine whether pyroptosis in macrophages plays a role in the initiation of liver IRI.

Whatever the mode of KC death, KCs that cannot sufficiently respond to their altered environments ultimately die (Guilliams and Scott, 2022). It is speculated that acutely induced disconnection of KCs with LSEC, HSCs, and hepatocytes due to liver IRI renders KCs incapable of maintaining their functional integrity (Bonnardel et al., 2019). It was also suggested that damaged LSECs and HSCs during liver IRI cannot secrete sufficient growth factors, including colony-stimulating-factor-1 (CSF1) and IL-34, to induce KC proliferation (Guilliams et al., 2020). Although the precise mechanisms of KC death during liver IRI remains to be elucidated, understanding these mechanisms represents an important question as the mode of death may have considerable consequences for how the liver subsequently responds to IRI.

### Replenishment of deleted KCs by MoMFs

KC depletion thorough cell death during acute liver injury induces recruitment of BM-derived monocytes to the injured regions to replace the hepatic macrophage population (Krenkel and Tacke, 2017; Guilliams and Scott, 2022) (Figure 1B). In mouse liver diseases, MoMFs are divided into two main subpopulations in terms of Ly6C expression levels: Ly6C^high^ and Ly6C^low^ MoMFs. Ly6C^high^ monocytes expressing C-C chemokine receptor type 2 (CCR2) are recruited by the CCR2 ligand CCL2 and is produced by KCs and HSCs. The recruited monocytes consist of a BM-derived pro-inflammatory Ly6C^high^ subset (McDonald and Kubes, 2016) that differentiate into pro-inflammatory MoMFs, which replace the resident macrophage population. Ly6C^high^ macrophages show pro-inflammatory properties with the expression of PRRs and chemokine receptors, including CCR2 that is a receptor for CCL2 (David et al., 2016). The CCL2/CCR2 axis mediates hepatic MoMF recruitment upon KC damage, leading to amplification of the inflammatory response in the injured liver. Pro-inflammatory MoMFs also produce inflammatory mediators, including cytokines and proteases, which digest damaged tissues to facilitate their clearance (Rahman et al., 2017). With the cessation of inflammatory responses, Ly6C^high^ macrophages differentiate into Ly6C^low^ macrophages that show reparative properties (David et al., 2016). Ly6C^low^ reparative macrophages contribute to the resolution of liver inflammation and restoration of damaged tissues induced by liver IRI.

In normal livers with genetically deleted KCs, MoMFs are thought to replace the vacant KC pool. Furthermore, recruited MoMFs following KC depletion display a phenotype shift toward that of the original resident KCs. In mouse models of selectively depleted *Clec4f*-expressing KCs, recruited monocytes replenished the liver macrophage population and differentiated toward a function similar to that of KCs within 1 month (Beattie et al., 2016; Scott et al., 2016; Bleriot and Ginhoux, 2019). In another model of conditional KC depletion, MoMFs acquired the resident KC profile within days of migration into the liver. The phenotype shift is caused by the concerted actions of MoMFs with LSECs, HSCs, and hepatocytes (Bonnardel et al., 2019; Sakai et al., 2019). Monocyte recruitment is primarily initiated by TLR signaling in KCs or HSCs, resulting in increased secretion of CCL2. KC deletion in *Clec4f*-*Dtr* mice activates HSCs and LSECs to release TNF-α and IL-1β, which upregulate monocyte-attracting chemokines, including CCL2. Additionally, the expression of adhesion molecules, including vascular cell adhesion molecule 1 and selectin E, are upregulated for the recruitment of monocytes (Bonnardel et al., 2019).

In IRI-stressed livers, BM-derived monocytes are recruited to replenish the hepatic macrophage population as a result of KC depletion (Yue et al., 2017; Nishizawa et al., 2018; Goto et al., 2021; Ni et al., 2021). Recruited BM-derived monocytes differentiate into pro-inflammatory MoMFs, which induce inflammation and contribute to disease progression. Loss of KCs and subsequent recruitment of MoMFs have also been demonstrated in other liver diseases. Reduction of the KC population was reported in models of APAP-induced liver injury (Zigmond et al., 2014), viral infection (Borst et al., 2018), and high fat diet-induced NASH (Daemen et al., 2021). The recruited pro-inflammatory MoMFs would then contribute to both liver damage and controlling damage. The proportion of replenished resident macrophages depends on the degree of depletion of resident macrophages. Extensive depletion of KCs would result in significant recruitment of MoMFs, whereas minimal depletion would induce a proportional response in the recruitment of MoMFs. Additionally, the degree of KC depletion appears to be dependent on the cause of liver pathology. Gene ablation induces extensive depletion of KCs in the liver, while APAP administration partially depletes KCs in the peri-central area of the liver. Furthermore, upon recruitment in response to liver injury, MoMFs differentiate into a variety of phenotypes with discrete functions depending on the microenvironment (Guilliams and Scott, 2022; Peiseler et al., 2022).

### Replenishment of KCs by other types of macrophages

Aside from the recruitment and differentiation of MoMFs in response to KC reduction, other macrophages have been suggested to accumulate in the injured areas to resolve liver inflammation and promote liver repair. In an experimental model of sterile focal thermal hepatic injury, mature large peritoneal macrophages expressing F4/80^+^CD11 b^+^GATA6^+^ crossed the mesothelium that covers the liver, leading to their accumulation at the site of hepatic injury within 1 h after insult. Recruitment of hepatic macrophages and subsequent liver repair was delayed in GATA6-deficient mice (Wang and Kubes, 2016). Interestingly, peritoneal macrophages migrated directly through the hepatic capsule, which is avascular, and did not pass through the microvasculature. Additionally, peritoneal macrophages that migrate through the hepatic capsule promote repair of damaged tissue not only on the surface of the liver created by the heat probe but also in the deeper regions of the liver induced by CCl_4_ administration. However, the fate of peritoneal macrophages that accumulated in the injured regions warrants further investigation. Although LCMs are recently identified subset of murine liver-resident macrophages, it is hypothesized that they sense peritoneal pathogens and recruit neutrophils to the liver capsule (Sierro et al., 2017). However, the roles of peritoneal macrophages and LCMs in liver inflammation and repair during liver IRI remain unknown.

In a diet-induced NASH model, the KC population was reduced due to cell death, preventing their proliferation and resulting in the recruitment of Ly6C^high^ monocytes. In mouse models of NASH, Ly6C^high^ monocyte-derived MoMFs differentiate into LAMs (Remmerie et al., 2020; Tran et al., 2020), which express the gene signatures associated with lipid metabolism including *Gpnmb, Spp1, Trem2,* and *Cd9* (Seidman et al., 2020) and localize in fibrotic areas (Daemen et al., 2021). However, a similar signature *(Gpnmb* and *Spp1)* was observed in macrophages around the bile-duct in healthy mouse livers (Guilliams et al., 2022). Furthermore, the transcriptional profiles of LAMs are also observed in recruited monocyte-derived macrophages in APAP-induced injured livers and appear to be distributed at sites of injury (Coelho et al., 2020; Daemen et al., 2021; Ben-Moshe et al., 2022). Because MoMFs clear cellular debris, including lipid-materials and liver macrophages that are involved in lipid metabolism (Bleriot et al., 2021), it may be plausible that these cells have a similar proflle to that of LAMs. Considering that LAMs are induced following efferocytosis (Doran et al., 2020) during the progression of NASH (Daemen et al., 2021), the accumulation of hepatic LAMs might inhibit liver fibrosis by removing damaged or dying cells and excessive lipid droplets. This also suggests that LAMs contribute to liver repair after acute liver injury. However, the presence of LAMs has not been confirmed in an IRI-stressed liver. Additionally, whether LAMs play distinct roles in liver IRI, represent distinct cell types of macrophages, or display plasticity during liver IRI remain unclear and warrant further investigation.

### Re-distribution of KCs

KC depletion leads to repopulation through MoMFs. In mice administered with APAP or CCl_4_, the KC population was transiently reduced but recovered to pre-administration levels upon resolution of liver inflammation, concomitant with the disappearance of MoMFs from liver tissues (Zigmond et al., 2014; Flores Molina et al., 2022). The phenotype of KCs was distinct from that of MoMFs during chemical-induced acute liver injury, and recovered KCs proliferated and regenerated. Despite the extensive reduction in the population of KCs in the peri-central area, it is speculated that the remaining KCs replenishes the KC pool during acute liver injury induced by APAP or CCl_4_. In agreement with this view, KCs in the repair phase of APAP-induced liver injury significantly upregulated genes related to proliferation, including *Mki67* and *Pcna* (Ben-Moshe et al., 2022).

In liver IRI, KC recovery was observed at 3 days post-reperfusion and further increased at 7 days post-reperfusion (Ni et al., 2021). However, recovered KCs lacked TIM-4. Other studies reported that although the KC population recovered at 96 h post-reperfusion, they were still lower than pre-reperfusion levels (60% reduction) and half of the recovered KCs appeared to originate from the BM (Nishizawa et al., 2018). Whether the population of restored KCs in the repair phase of liver IRI are similar to the original KCs or whether they will give rise to original KCs remains to be elucidated. Recent studies have suggested that recovered KCs display an immune-suppressive role during acute liver injury. The restored KCs after acute APAP-induced liver injury exhibited impairment in bacterial clearance, which was mediated by interactions with KCs and T cells through the programmed cell death-1 (PD-1) and PD-1 ligand 1 pathway (Triantafyllou et al., 2021). Thus, the function of regenerated KCs following liver IRI warrants further investigation.

### Macrophage reprogramming

Differentiated macrophages that express Ly6C^high^ from Ly6C^high^ monocytes predominate the population of macrophages in the early phase of acute liver injury induced by IRI (Yue et al., 2017; Nishizawa et al., 2018; Goto et al., 2021). In response to the initial inflammation and to control damage, macrophages transmit signals for the resolution of inflammation, removal of debris, and initiation of tissue repair. To repair damaged tissue, phenotype switching of Ly6C^high^ pro-inflammatory macrophages to Ly6C^low^ reparative macrophages at the sites of injury is crucial (Ramachandran et al., 2012; Krenkel and Tacke, 2017). *In situ* reprogramming of macrophages has been demonstrated using intravital microscopic analysis (Dal-Secco et al., 2015). Recruitment of Ly6C^high^ monocytes to the damaged areas is induced by localized sterile thermal injury on the surface of the liver. The migrant Ly6C^high^/CCR2^+^ monocytes subsequently transform into Ly6C^high^ MoMFs. Afterwards, Ly6C^high^ MoMFs differentiate into Ly6C^low^ reparative macrophages *in situ*. Despite spleen-derived MoMFs being characterized as Ly6C^low^CCR2^low^CX3CR1^high^, *in vivo* tracking analyses demonstrated that spleen-derived CCR2^low^CX3CR1^high^ monocytes failed to infiltrate the liver. Selective ablation of Ly6C^high^ monocytes, and consequently of their MoMF descendants, impaired the repair and regeneration of the liver from APAP-induced liver injury, suggesting their critical role in the resolution and repair of liver damage (Zigmond et al., 2014; Graubardt et al., 2017). For macrophage phenotype switching, anti-inflammatory cytokines, including IL-4 and IL-13, are involved (Bosurgi et al., 2017). Additionally, Ly6C^low^ macrophages switch to the restorative phenotype by expressing growth factors, matrix metalloproteinases (MMPs), and phagocytosis-related genes (MARCO), which facilitate tissue repair and restoration. Thus, the phenotype shift of macrophages from a pro-inflammatory to a reparative phenotype in the damaged liver is crucial for liver repair following IRI (Nishizawa et al., 2018; Ye et al., 2020). Although reparative macrophages are generated *in situ* through phenotype switching of pro-inflammatory macrophages, it remains unclear whether reparative macrophages are also recruited during the repair phase of liver diseases.

The functional roles of pro-inflammatory macrophages in liver repair after acute liver injury appear to be context-dependent. It was suggested that pro-inflammatory macrophages worsen inflammation by secreting pro-inflammatory mediators, further worsening tissue injury. Extensive inflammation induced by pro-inflammatory macrophages aggravates inflammation (Ramachandran et al., 2012) and does not repair tissues. For tissue restoration, pro-inflammatory macrophages also produce inflammatory mediators, including cytokines and proteases, that clear dead cells, digest the extracellular matrix, and resolve inflammation (Nahrendorf et al., 2007; Rahman et al., 2017). As macrophages have been classified by the over-simplified macrophage polarization, these findings may suggest the presence of transition macrophages during the process of macrophage differentiation (Dal-Secco et al., 2015).

The clearance of dead cells by macrophages, a process termed efferocytosis, is critical for the resolution of inflammation and maintenance of tissue homeostasis (Doran et al., 2020). Efferocytosis is also a key process in macrophage phenotype switching that initiates the resolution phase of inflammation (Soehnlein and Lindbom, 2010). The phagocytosis of apoptotic neutrophils by macrophages stimulates macrophage phenotype switching toward the reparative phenotype, which then promotes tissue repair in APAP-induced acute liver injury in mice (Triantafyllou et al., 2018a). Meanwhile, ablation of Ly6C^low^ macrophages increases levels of ROS-producing apoptotic neutrophils, leading to persistent APAP-induced liver injury (Graubardt et al., 2017). Additionally, impaired macrophage phenotype switching by efferocytosis sustains ROS-producing apoptotic neutrophils and inflammation. Phagocytosis of apoptotic neutrophils under the presence of anti-inflammatory cytokines, including IL-4 or IL-13, induces *in situ* macrophage reprogramming (Bosurgi et al., 2017). IL-4 and IL-33 derived from necrotic KCs shifts the macrophage phenotype from pro-inflammatory Ly6C^high^ to reparative Ly6C^low^ (Bleriot et al., 2015). When apoptotic cells upregulate the expression of phosphatidylserine on their outer plasma membrane, macrophages expressing T cell immunoglobulin and TIM-4 recognize and bind phosphatidylserine directly to induce phagocytosis. In addition to efferocytosis of Ly6C^low^ macrophages, KC efferocytosis, which is regulated by the efferocytosis receptor TIM-4, contributes to the resolution of inflammation induced by liver IRI (Ni et al., 2021). Another efferocytosis receptor, c-mer proto-oncogene tyrosine kinase (MerTK), is involved in macrophage polarization to the reparative phenotype to repair tissues damaged by liver IRI (Zhang et al., 2023).

### Pro-resolving factors from reparative macrophages

Reparative macrophages have the potential to support the resolution of inflammation through the secretion of regenerative growth factors and anti-inflammatory cytokines. In the resolution of liver inflammation and fibrosis induced by carbon tetrachloride (CCl_4_) administration in mice, reparative macrophages upregulate the expression of genes related to the production of growth factors, including insulin-like growth factor 1, and phagocytosis, including MerTK. Additionally, reparative macrophages also activate triggering receptor expressed on the myeloid cells 2 (TREM2) encoding an innate immunity scavenger receptor implicated in phagocytosis and clearance of apoptotic cells, and upregulate genes related to matrix degradation, including *Mmp12* (Ramachandran et al., 2012). The converted Ly6C^low^ macrophages promote tissue repair by inducing angiogenesis *via* VEGF-A, allowing for the reconstruction of the extracellular compartment, phagocytosis, and disposal of dead cells (Zigmond et al., 2014; Stutchfield et al., 2015) during APAP-induced liver injury. To promote liver repair after IRI, recruited macrophages also secrete pro-reparative factors that contribute to tissue regeneration *via* EGF (Ohkubo et al., 2014). Meanwhile, IL-10 generated from KCs protect the liver against IRI and suppress the progression of liver inflammation. Exogenous administration of IL-10 attenuated IRI in KC-depleted livers due to clodronate liposome administration (Ellett et al., 2010). KCs also resolved liver inflammation due to IRI *via* TIM4-mediated IL-10 upregulation (Yue et al., 2017). Regarding the fate of reparative macrophages following liver IRI, further studies are required to determine whether their population is reduced upon recovery from IRI or they give rise to liver-resident macrophages to maintain the KC pool under steady state environments.

### Interactions of MoMFs with other hepatic immune cells

Aside from efferocytosis, the interactions of other hepatic immune cells with recruited MoMFs are essential for macrophage phenotype switching after liver IRI (Figure 1B).

***Dendritic cells*** (***DCs***) ***.*** During the repair phase following liver IRI in mice, monocyte-derived DCs (mo-DCs) mediate switching of the macrophage phenotype from pro-inflammatory to pro-reparative by producing IL-13, which in turn resolves liver inflammation and promotes liver repair (Nakamoto et al., 2020). *In vitro* co-culture experiments have shown that IL-13 facilitates the transition from Ly6C^high^ macrophages to Ly6C^low^ macrophages as well as increased IL-10 production by macrophages. These results suggest that crosstalk between mo-DCs and macrophages stimulates liver tissue repair after acute injury.

***Invariant natural killer T*** (***iNKT***) ***cells***. iNKT cells recognize glycolipid antigens expressed on the surface molecule, CD1d, by the invariant T cell receptor (Van Kaer, 2007). iNKT cells constitute 30% of all lymphocytes and are frequently found in the liver (Crosby and Kronenberg, 2018) where they patrol sinusoids constantly in a random fashion (Geissmann et al., 2005). Upon activation, iNKT cells secrete large amounts of cytokines, including interferon (IFN)-γ and IL-4, as well as chemokines that modulate subsequent immune responses (Van Kaer, 2007; Crosby and Kronenberg, 2018). iNKT cells play a critical role in the promotion of liver tissue repair after focal sterile thermal injury through monocyte transition from an inflammatory to a reparative phenotype *via* IL-4 (Liew et al., 2017). Furthermore, activated iNKT cells facilitate liver recovery through the acceleration of macrophage phenotype switching from a pro-inflammatory to a reparative phenotype in the early phase of liver IRI (Goto et al., 2021). Activated iNKT cells were shown to produce both IL-4 and IFN-γ through interaction with CD1d in macrophages. IFN-γ-induced accumulation of pro-inflammatory macrophages and IL-4-induced macrophage phenotype switching contribute to the repair of tissues following liver IRI (Goto et al., 2021). Consistent with these findings, iNKT cells also contribute to the resolution of inflammation and tissue repair after IRI in the heart (Sobirin et al., 2012). These findings indicate that iNKT cells interact with macrophages to facilitate the resolution of hepatic inflammation and repair after acute liver injury. Meanwhile, iNKT cell activation inhibits liver regeneration after partial hepatectomy in mice, which is mediated through the secretion of both IFN-γ and IL-4 from increased numbers of iNKT cells (Yin et al., 2014). The importance of the KC/NKT cell interaction in liver regeneration has also been described. After partial hepatectomy, MoMFs produce IL-12 to activate hepatic NKT cells, which prohibit liver regeneration (Wu et al., 2015). These discrepant results may be due to the difference in the experimental models. Liver IRI models contain severe injury components that are associated with the infiltration of inflammatory cells, including recruited innate immune cells. In contrast, partial hepatectomy models have minimal liver injury that is associated with the proliferation of resident liver macrophages but not with the accumulation of inflammatory cells.

Group 2 innate lymphoid cells stimulated with IL-33 alleviate liver IRI through IL-4-mediated macrophage shifting toward a reparative phenotype that expresses CD11 b^+^F4/80^high^CD206^high^ in mice (Zhang et al., 2022).

***Neutrophils.*** Regarding liver IRI, intense infiltration of neutrophils and dysregulation of neutrophil activity that leads to excessive amounts of proteinases and ROS production can aggravate tissue damage (Jaeschke et al., 1990). Significant infiltration of activated neutrophils causes excessive tissue destruction and persistent inflammation, which prevents repair following liver IRI. On the other hand, neutrophils also facilitate the recovery from tissue injury by producing lytic enzymes and ROS necessary for the clearance of damaged tissue and necrotic cells (Bratton and Henson, 2011). Neutrophils accumulate in injured tissues to remove tissue debris, indicating that neutrophils act as phagocytes for tissue repair (Wang et al., 2017). Activated neutrophils are recruited into the liver in the late phase of APAP-induced liver injury to remove cell debris for tissue regeneration (Williams et al., 2014). Therefore, the relevance of neutrophils in any setting is context-dependent, and timely termination of neutrophil activity and their clearance appear to be essential for the resolution of liver injury. Interestingly, during APAP-induced liver injury, depletion of Ly6C^high^ monocytes, and consequently of reparative macrophages, with CCR2 blockade induced substantial accumulation of ROS-producing apoptotic neutrophils, which can lead to liver injury and impair liver repair (Graubardt et al., 2017). These findings suggest that reparative macrophages may facilitate injury resolution and liver repair after acute liver injury by clearing apoptotic neutrophils. Additionally, neutrophil depletion during the recovery phase of CCl_4_-induced liver injury prolonged liver inflammation and impaired inflammation resolution by interfering with hepatic macrophage phenotype switching (Calvente et al., 2019). Furthermore, neutrophil-derived ROS triggers macrophage phenotype switching to a reparative phenotype to promote liver repair after APAP-induced acute liver injury (Yang et al., 2019). Moreover, neutrophil-derived lipocalin stimulates the recruitment of pro-inflammatory macrophages that promote the clearance of debris and apoptotic cells induced by myocardial IRI, leading to injury resolution and cardiac repair (Horckmans et al., 2017). In a hepatic focal sterile heat injury model, neutrophils not only migrated into the damaged hepatic microvasculature but also created tunnels for new microvascular regrowth, probably induced by MMP-9 and VEGF from neutrophils (Wang et al., 2017; Hossain and Kubes, 2019), which in turn promoted liver repair. Although accumulating evidence has uncovered neutrophil pro-reparative capabilities, little is known about neutrophil functions in liver IRI (Nakamura et al., 2019) and crosstalk of macrophages with neutrophils for inflammation resolution and tissue repair after liver IRI.

***T cells.*** Although CD4^+^ T cells have been implicated in liver IRI, the interaction of T cells with macrophages mediated by inducible T cell costimulatory (ICOS) and its ligand (ICOSL) contribute to liver repair after CCl_4_-induced acute liver injury (Ramavath et al., 2021). CD8^+^ T cells expressing costimulatory molecule ICOS that interact with MoMFs expressing ICOSL promote MoMF phenotype switching to reparative macrophages, which co-express TREM-2 and MerTK, contributing to efferocytosis. In an acute lung injury mouse model, IL-13 derived from regulatory T cells stimulated macrophages to produce IL-10, which triggered the resolution of inflammation by enhancing macrophage efferocytosis (Proto et al., 2018). These studies suggest that crosstalk between macrophages and T cells or Treg cells orchestrates the removal of apoptotic cells to stimulate liver repair after liver IRI.

### Possible therapeutic strategies for targeting macrophages in liver IRI

As hepatic macrophages contribute to liver repair and regeneration after liver IRI, therapies that promote a switch from a pro-inflammatory phenotype to a restorative phenotype would be a beneficial approach to accelerate inflammation resolution and promote liver repair. Meanwhile, macrophages markedly show heterogeneity with contrasting functions in the initiation and progression of liver diseases, and the development of strategies targeting pathologic macrophage phenotypes without affecting the functions of other phenotypes is hampered. Additionally, a disparity in macrophage phenotypes between mice and humans hampers the application of translational studies in mice to therapeutic options for human patients. Nevertheless, therapeutic strategies for targeting hepatic macrophages have been delineated (van der Heide et al., 2019). Although there are several approaches for targeting hepatic macrophages, we review the therapeutic approaches to macrophage reprogramming that utilize agents and targeted cells for the promotion of liver inflammation resolution and repair after acute liver injuries, including liver IRI.

Macrophage-modulating biomaterials and biologics have been used as agents for skewing macrophage differentiation into the restorative phenotype (Triantafyllou et al., 2018b; van der Heide et al., 2019).

Macrophage phenotype transition toward the reparative phenotype is mediated by anti-inflammatory cytokines, including IL-4, IL-10, and IL13 (Ruytinx et al., 2018). Anti-IL-4 antibody treatment delayed liver repair by increasing levels of pro-inflammatory macrophages during liver IRI (Goto et al., 2021). Blockade of IL-4/IL-10 also impaired the resolution of liver inflammation and suppressed macrophage polarization in a sterile hepatic focal thermal injury model (Liew et al., 2017). IL-13 derived from Tregs increased macrophage efferocytosis through IL-10 (Proto et al., 2018). IL-13 levels were increased during the reparative phase of liver IRI as well as due to IL-13-induced macrophage polarization in cultured BM-derived MFs (Nakamoto et al., 2020). IL-10 produced by Tregs has been implicated in attenuating liver inflammation and promoting inflammation resolution.

Secretory leukocyte protease inhibitor, an anti-inflammatory protein identified in mice and humans with acute liver failure (Antoniades et al., 2014), facilitates differentiation toward MerTK-positive restorative macrophages. Because MerTK is a phagocytic receptor that recognizes apoptotic cells, MerTK-positive macrophages promote resolution of liver inflammation and liver repair in acute liver failure by enhancing the clearance of apoptotic neutrophils (Triantafyllou et al., 2018a). Similarly, macrophage CSF1 also improves liver repair from APAP-induced acute liver failure through the transformation of infiltrated Ly6C^high^ macrophages into Ly6C^low^ phagocytes at the site of liver injury (Stutchfield et al., 2015). Furthermore, CSF1 promotes liver recovery from liver IRI and is associated with increased levels of macrophages, which are presumably reparative, during the repair phase of liver IRI (Konishi et al., 2020).

Glycogen synthase kinase *β* (Gskβ) regulates tissue inflammation. In murine models of liver IRI, genetic deletion of Gsk3β or pharmacological inhibition of Gsk3 stimulates recovery from acute liver injury by increasing the expression of pro-resolution properties in infiltrating macrophages (Zhang et al., 2023). Gsk3β inactivation decreases the expression of genes related to pro-inflammatory macrophages, increases the expression of genes related to reparative macrophages, and induces efferocytosis in macrophages.

Neutrophil-derived extracellular vesicles containing microRNA-223 (miR-223) favors macrophage polarization toward a reparative phenotype and promotes liver repair in mice administered with CCl_4_ (Calvente et al., 2019). As non-coding micro-RNAs show potential in treating acute liver injury, exogenous microvesicular delivery of miR-223 appears to facilitate injury resolution and repair after liver IRI by macrophage polarization.

Netrin-1 was identified as a neuronal guidance cue directing neuronal axons to targets during the development of the nervous system. Additionally, netrin-1 was shown to be an immune mediator. In liver IRI models, netrin-1 promotes liver inflammation resolution and liver repair by controlling the infiltration of Ly6C^low^ macrophages (Schlegel et al., 2016).

Prostaglandin E_2_ (PGE_2_) is synthesized by microsomal PGE synthase-1 (mPGES-1) during liver IRI. Although PGE_2_ exhibits dual roles in inflammation, deletion of mPGES-1 or the mPGES-1 inhibitor facilitated liver repair after liver IRI by promoting macrophage phenotype switching from a pro-inflammatory to a reparative type. One of the receptor subtypes for PGE_2_ in macrophages, namely, EP4, seems to be involved in macrophage plasticity (Nishizawa et al., 2018).

Another strategy to induce macrophage polarization is by promoting signaling through the peroxisome proliferator-activated receptor (PPAR) pathway. PPARs are nuclear transcription factors essential for liver homeostasis, lipid metabolism, and inflammation. PPAR-γ agonists mitigate liver IRI during the injury phase by decreasing levels of polarized pro-inflammatory macrophages (Ding et al., 2021). In contrast, PPAR agonists do not affect the infiltration of hepatic MoMFs after acute liver injury induced by CCl_4_ administration (Lefere et al., 2020).

The transfer of exogenous macrophages would represent an alternative approach by inducing phenotype switching for liver repair and regeneration following liver IRI. Cell-based therapies transferring *ex vivo* differentiated autologous macrophages have been reported in experimental animals and in patients with liver cirrhosis (Starkey Lewis et al., 2019). In APAP-induced liver injury, adoptive transfer of *ex vivo* IL-4/IL-13-polarized BM-derived macrophages attenuated liver injury and promoted the proliferation of hepatocytes and LSECs (Starkey Lewis et al., 2020), indicating that infusion of polarized reparative macrophages may be a therapeutic option for the treatment of acute liver injury. Clinical trials in humans for the safety and efficacy of autologous macrophage therapy in compensated liver cirrhosis are currently underway (Moroni et al., 2019). Regarding liver IRI, HO-1 expression in macrophages reduced liver inflammation by promoting macrophage polarization toward the reparative phenotype. In human liver transplantation biopsies, higher HO-1 levels were correlated with lower levels of pro-inflammatory markers and higher levels of anti-inflammatory markers (Zhang et al., 2018). Correspondingly, adoptive transfer of HO-1 overexpressing BM-derived macrophages mitigated IRI in orthotopic liver transplantation (OLT) in mice. In liver samples from human OLT, high levels of HO-1 were associated with low levels of alanine transaminase (Nakamura et al., 2018).

Almost all of the above studies on immune modulation strategies for targeting macrophage activity rely on the use of experimental animal models and *in vitro* studies. Therefore, the relevance of these findings from the preclinical studies must be validated in humans. The human clinical trials would lead to the development of better therapeutic approaches to macrophage reprogramming for promoting liver repair after liver IRI.

## LSECs in liver repair

**FIGURE 2:**
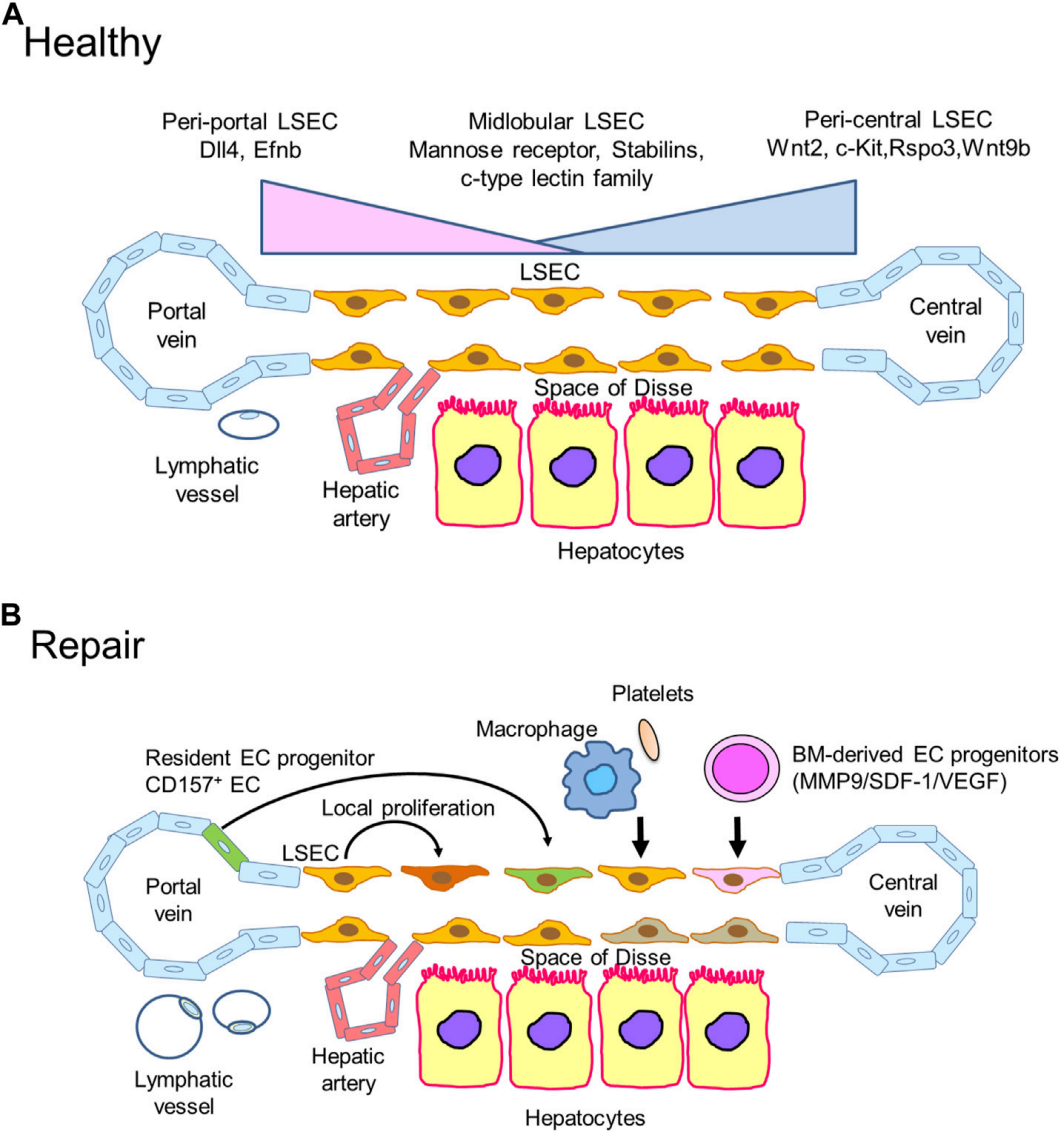
Liver sinusoidal endothelial cells (LSECs) in liver repair **(A)**. Zonation of LSECs. LSECs secrete specific angiocrine factors in a zonation-specific manner from the portal vein to the central vein. Peri-central LSECs modulate the spatial division of hepatocytes by secreting angiocrine Wnt ligands and Wnt-signaling enhancer RSPO3. Hepatic lymphatic fluids mainly drain into the lymphatic vessels in the portal tract. **(B)**. During acute liver injury, including liver IRI, damaged LSECs are restored and re-renewed through different mechanisms. The proliferation of remaining neighboring LSECs replaces lost LSECs by producing angiocrine factors, including hepatocyte growth factor (HGF), Wnt, and stem cell factor (SCF). Accumulated reparative macrophages and platelets participate in restoring LSECs in the injured area by releasing LSEC growth factors. BM-derived EC progenitors are recruited to repair damaged LSECs through the stromal cell-derived factor-1 (SDF-1)/CXCR7 axis. Additionally, resident EC progenitors expressing CD157 adjacent to the portal vein contribute to LSEC regeneration after severe LSEC damage. The formation of lymphatic vessels around the portal tract in response to liver IRI promotes liver repair by clearing necrotic tissue debris.

Liver sinusoids are lined by LSECs, which represent approximately 15%–20% of liver cells. LSECs constitute a unique microvascular bed of the liver that regulate liver inflammation and immunity (Shetty et al., 2018). LSECs also serve powerful scavenger functions by clearing macromolecular waste molecules from the circulation (Bhandari et al., 2021). LSECs play an important role in the maintenance of liver homeostasis by secreting angiocrine factors (Kostallari and Shah, 2016). Furthermore, recent advancements in single-cell technology have revealed the heterogenous distribution of LSECs (Halpern et al., 2018). Upon injury to LSECs, regeneration is achieved by local proliferation of adjacent LSECs and endothelial progenitor cells derived from the BM or peri-portal niche (Figure 2).

### LSEC heterogeneity and zonation in healthy livers

Recent developments in single-cell technology demonstrate the heterogeneity of liver ECs, including LSECs, in mice (Halpern et al., 2018) and humans (MacParland et al., 2018; Aizarani et al., 2019) (Figure 2A). The spatial and molecular changes in gene expression along the axis of the liver lobule from the portal to the central vein are referred to as liver zonation (Halpern et al., 2017). According to liver zonation in hepatic sinusoids, LSECs are categorized into peri-central, mid-lobular, and peri-portal subtypes. The specialized angiocrine factors from LSECs in different zonations maintain their integrity and control adjacent hepatocytes. Particularly, liver zonation is controlled by peri-central LSEC-derived signals involving Wnt factors (Leibing et al., 2018) and the Wnt-signaling enhancer R-spondin-3 (RSPO3) (Rocha et al., 2015). Single-cell RNA sequence and spatial transcriptomic analyses revealed enriched expression of *Wnt2, Wnt9b, Rspo3,* and *c-kit* in peri-central LSECs (Halpern et al., 2018). The Wnt ligands Wnt2 and Wnt9 play a critical role in hepatocyte proliferation. The angiopoietin receptor Tie-1 shapes LSEC zonation and regulates *Wnt9b* expression in LSECs (Inverso et al., 2021). Mid-lobular LSECs represent the major EC subpopulation and express specialized scavenger receptors such as the mannose receptor, stabilins, and c-type lectin family (Bhandari et al., 2021). Single-cell and spatial transcriptomic analyses also demonstrated that peri-portal LSECs are enriched in genes related to vessel development, including delta-like canonical Notch ligand 4 (*Dll4*), a ligand for Notch1 and *Efnb2* (Halpern et al., 2018). These findings indicate that LSECs secrete specific angiocrine factors to maintain LSEC homeostasis by self-renewal in a zonation-specific manner from the portal to the central vein (Gomez-Salinero et al., 2021). LSEC zonation in the healthy human liver has also demonstrated upregulated expression of *LYVE-1* and *FCN3* in peri-central and mid-lobular LSECs (Aizarani et al., 2019).

### LSEC repair and regeneration

After the liver is damaged by acute insults, including hepatotoxicants, liver resection, and liver IRI, damaged LSECs need to regenerate (Figure 2B). Reconstruction of the liver microvasculature provides sufficient blood and nutrients, contributing to liver repair. Thus, LSEC repair and regeneration to restore blood supply are critical events during the repair phase of acute liver injury. Indeed, LSEC loss is associated with impaired liver regeneration in patients with acute-on-chronic liver injury (Shubham et al., 2019).

The formation of blood vessels is thought to result from the expansion of endothelial cells comprising neighboring vessels (Chung and Ferrara, 2011). Recent studies have shown how zonal LSEC subpopulations support the regeneration process of LSEC and hepatocytes in response to acute liver injury (Ben-Moshe et al., 2022). In mice treated with APAP, injured peri-central LSECs downregulated the expression of *Wnt2, Wnt9b*, and *Rspo3* during the injury phase of APAP toxicity, which were then upregulated in the repair phase of APAP hepatotoxicity. Wnt9 from peri-central LSECs plays an important role in liver regeneration after partial hepatectomy (Preziosi et al., 2018). LSECs in the mid-lobular and peri-central regions markedly displayed enhancement of proliferation genes including *Mki67*, suggesting that dead LSECs in peri-central regions were replaced through the proliferation of remaining neighboring cells. Meanwhile, hepatocyte growth factor (*Hgf*) expression was enhanced in all peri-portal, mid-lobular, and peri-central LSECs, indicating that *Hgf* upregulation in LSECs influences hepatocyte proliferation in all regions during the repair phase of APAP toxicity. Additionally, late administration of the Wnt agonist FL6.13 stimulated liver repair and regeneration after APAP toxicity (Hu et al., 2022). Because vascular Wnt ligands in peri-central LSECs are regulated by Tie1 signaling, Tie1 blockade impaired liver regeneration after partial hepatectomy *via* reduced levels of Wnt2 and Wnt9b (Inverso et al., 2021). Additionally, liver regeneration after partial hepatectomy was also facilitated by Wnt2 production in mid-lobular and peri-central LSECs expressing c-kit (Duan et al., 2022). The transfer of c-kit^+^ LSECs mitigated CCl_4_-induced peri-central liver necrosis associated with increased proliferation of peri-portal hepatocytes, suggesting that activation of the stem cell factor and its receptor, c-kit pathway in LSECs contributes to liver repair after acute liver injury. Therapeutic strategies for targeting LSEC zonated genes may facilitate liver repair and regeneration after acute liver injury.

LSEC-derived angiocrine paracrine-acting cytokines also regulate the regenerative functions of stem cells in response to liver damage. Certain EC populations, including BM-derived EC progenitor cells, participate in the restoration of the population of LSECs and promotion of liver regeneration after monocrotaline-induced LSEC injury (Harb et al., 2009) or partial hepatectomy (Wang et al., 2012). The recruitment of BM-derived EC progenitors is mediated by the stromal cell-derived factor-1/CXCR7 axis (DeLeve et al., 2016).

Additionally, LSEC repopulation after monocrotaline-induced liver injury and subsequent radiation did not require BM-derived progenitor cells but resident EC progenitors residing adjacent to the portal vein (Wakabayashi et al., 2018). The LSEC progenitors originating from the portal veins were characterized as CD157/CD200 double-positive endothelial cells, which migrated into the hepatic sinusoids to replace the damaged LSECs and proliferated.

In a model of liver IRI, LSECs are highly susceptible during cold and warm preservation (Teoh et al., 2007; Russo et al., 2012). Liver IRI-induced LSEC injury is of interest as it is characterized by the disruption of fenestrations and formation of large gaps, which is quite similar to that in APAP- or monocrotaline-induced acute liver injury, and MMP-9 activation is involved in the gap formation in LSECs (McCuskey, 2008). Therefore, as mentioned above, it is plausible that LSEC restoration from liver IRI may be achieved by the local proliferation of adjacent LSECs and BM-derived or resident EC progenitor cells. Indeed, hepatic inhibition of MMP-9 reduced early liver IRI and was associated with the mobilization of BM-derived EC progenitors to repair damaged LSECs in rats (Wang et al., 2020). Further studies are required to examine changes in the zonal distribution (genetic landscapes) of LSECs and the appearance of disease-associated LSEC populations and their functional roles in liver IRI.

### LSEC repair through interaction with platelets

Although the sequestration of platelets in hepatic sinusoids contributes to the progression of liver IRI (Teoh et al., 2007), platelet-derived pro-angiogenic mediators, including HGF, insulin-growth factor-1, and VEGF, contribute to the proliferation and regeneration of LSECs, leading to liver repair and regeneration. During liver regeneration after partial hepatectomy, direct contact of platelets with LSECs triggers the production of IL-6 (a key hepatocyte mitogen) from LSECs (Liang et al., 2022; Morris and Chauhan, 2022). Accumulation of platelets in hepatic sinusoids mitigates LSEC damage and helps regenerate damaged LSECs, resulting in liver regeneration (Meyer et al., 2015). The sequestration of platelets in hepatic sinusoids treated with monocrotaline attenuated LSEC injury through VEGF secretion from platelets and stimulated liver repair (Otaka et al., 2020). These observations suggest that adhering platelets to the hepatic sinusoids release growth factors to repair the injured LSECs and promote liver repair after liver IRI.

### LSEC repair through interaction with MoMFs

The interaction of macrophages with LSECs also contributes to the resolution of inflammation and liver repair from acute liver injury. Deletion of monocytes/macrophages causes a delay in liver regeneration after partial hepatectomy and suppression of vascular growth in mice (Melgar-Lesmes and Edelman, 2015). In mice with APAP-induced liver injury, deletion of TREM-2, which is a regulator of inflammation, suppressed liver repair with the suppression of macrophage polarization and increased populations of damaged LSECs (Coelho et al., 2020). VEGF-A/VEGFR1 signaling in macrophages promotes liver repair and sinusoidal reconstruction after liver IRI *via* EGF production from macrophages (Ohkubo et al., 2014). During liver regeneration induced by partial liver resection, macrophages, in addition to LSECs, express several Wnt ligands, including Wnt2 and Wnt9b, which stimulate *β*-catenin signaling in hepatocytes to promote hepatocyte proliferation (Preziosi et al., 2018). These findings suggest that LSEC repair through interaction with infiltrative MoMFs facilitates liver repair after acute liver injury, including liver IRI.

## Lymphatic vessels in liver repair

The hepatic vascular system includes the hepatic microvascular system as well as the lymphatic vascular system. Liver ECs (liver vascular endothelium) are composed of LSECs as well as liver lymphatic ECs (lymphatic vessels). Recent studies have revealed the roles of lymphatic vessels in health and diseased livers, including liver cirrhosis, tumors, liver transplantation, and NASH (Jeong et al., 2022). Under physiological conditions, hepatic lymphatic vessels localize in the space of Disse, which is an interstitial space between LSECs and hepatocytes. Hepatic lymphatic fluids contain sinusoidal plasma, and lymphatic fluids collect in the space of Mall and drain into the lymphatic ducts along the portal vein (Bobe et al., 2022). Thus, hepatic lymphatic vessels participate in the maintenance of fluid homeostasis. The formation of the peri-natal hepatic lymphatic vasculature is mediated by the VEGF-C/VEGFR3 signaling pathway (Bobe et al., 2022).

The quiescent state of hepatic lymphatics can be disrupted by pathological conditions such as acute or chronic hepatic inflammation, resulting in lymphangiogenesis and lymphatic vessel remodeling. CCl_4_-induced cirrhosis in rats resulted in the proliferation of hepatic lymphatic vessels (Vollmar et al., 1997). The number of lymphatic vessels around the portal tract was also increased in diet-induced NASH mice and in patients with NASH (Burchill et al., 2021). However, the transcript gene expression related to lymphatic ECs, including lymphatic vessel endothelial hyaluronan receptor 1, VEGFR3, prospero homeobox protein 1, and podoplanin, was reduced, suggesting a dysfunction of lymphatic vessels. This condition was also associated with impaired lymphatic drainage, and the therapeutic efficacy of VEGF-C administration on lymphangiogenesis and resulting enhanced lymphatic drainage has been shown in mice with NASH.

In a rat liver transplantation model, enhanced lymphangiogenesis in the graft was associated with the survival of recipients, and diminished lymphangiogenesis was found in areas of acute graft rejection (Ishii et al., 2010). Hepatic lymphangiogenesis may attenuate acute immune-cellular responses to liver transplantation. During liver IRI in mice, lymphatic vessels around the portal veins expanded and enlarged through VEGFR3 signaling (Nakamoto et al., 2020) (Figure 2B). Meanwhile, blockade of VEGFR3 delayed liver repair after liver IRI and suppressed lymphangiogenesis and lymphatic drainage. Exogenous administration of VEGF-D facilitated liver repair and attenuated liver IRI. Furthermore, recruited macrophages expressing VEGFR3 contributed to the formation of lymphatic vessels during liver repair after liver IRI. Expanded lymphatic vessels around the portal veins in response to liver IRI facilitates liver repair presumably by enhancing clearance of necrotic tissue debris and fluids. Similarly, the therapeutic potential of enhancing lymphangiogenesis for cardiac repair after myocardial infarction has been reported (Henri et al., 2016). Particularly, efferocytosis of macrophages in the infarcted regions induces VEGF-C production, promoting cardiac lymphangiogenesis and repair after myocardial ischemia (Glinton et al., 2022). However, some studies failed to demonstrate the beneficial role of increased lymphangiogenesis in cardiac repair and functional recovery from myocardial infarction and congestive heart failure (Keller et al., 2021). More studies are necessary to investigate whether lymphatic vessels and lymphatic drainage may be potential therapeutic targets for inflammation resolution and liver repair after liver IRI.

## HSCs in liver repair

**FIGURE 3:**
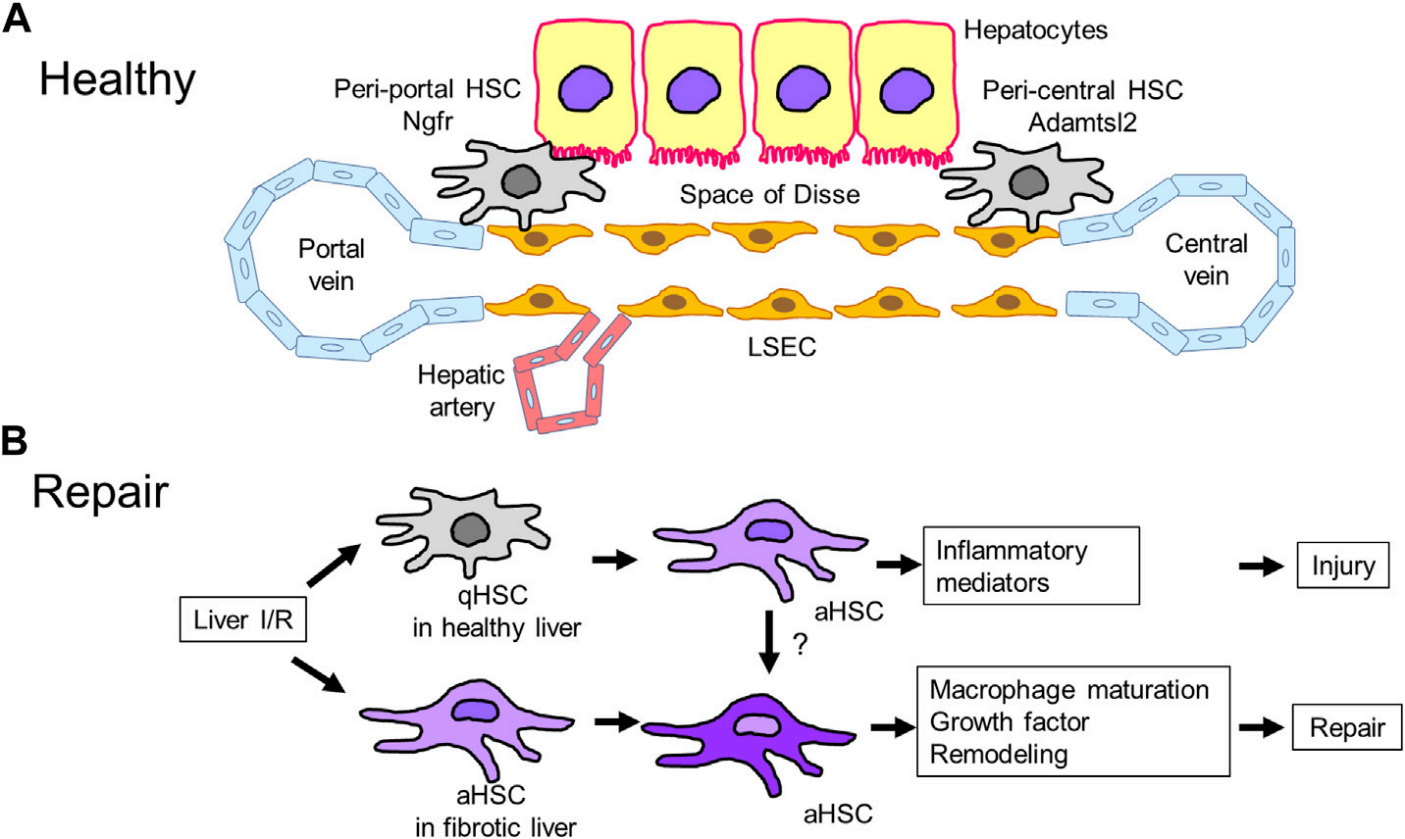
Hepatic stellate cells (HSCs) in liver repair **(A)**. HSCs reside in the space of Disse, which is a perisinusoidal space between LSECs and hepatocytes. HSCs in healthy livers include two subpopulations: peri-portal and peri-central HSCs. Peri-portal HSCs express nerve growth factor receptor (Ngfr), and peri-central HSCs express Adamts like 2 (Adamtsl2). **(B)**. Liver IRI induces HSC transition from quiescent to activated HSCs, leading to the development of liver injury through the release of inflammatory mediators. Meanwhile, livers with IRI-induced fibrosis containing activated HSCs minimize injury and stimulate repair associated with macrophage polarization, remodeling by proteinase production, and release of growth factors, but the underlying mechanisms are currently unidentified. aHSC, activated HSC; qHSC, quiescent HSC.

HSCs are localized in the space of Disse between hepatocytes and endothelial cells. The HSC population comprises approximately 5%–8% of the resident parenchymal cells. At a resting state, quiescent HSCs store vitamin A. When the liver is injured, activated HSCs trans-differentiate into myofibroblasts. Myofibroblasts are proliferative, inflammatory, contractile, and chemotactic. In addition, myofibroblasts play a central role in extracellular matrix remodeling and fibrosis. The conversion of HSCs to myofibroblasts is regulated by several signaling molecules and pathways. The key signaling pathways include the transforming growth factor-beta and platelet-derived growth factor pathways, which contribute to the development of liver fibrosis. In addition to these signaling pathways, the Hedgehog signaling pathway and innate immune signaling mediated by TLRs and cytokines also implicate HSCs activation (Tsuchida and Friedman, 2017). During liver IRI, HSCs play a role in the development of liver inflammation. HSC-depleted mice exhibited attenuation of liver IRI due to reduced TNFα levels and neutrophil infiltration (Stewart et al., 2014), suggesting that HSCs induce liver inflammation by producing pro-inflammatory mediators. Although the role of HSCs in the process of liver repair after IRI remains uncertain, some studies have shown that activated and proliferated HSCs contribute to liver repair by generating an extracellular matrix (Konishi et al., 2019), which was associated with increased levels of reparative macrophages in fibrotic livers following liver IRI. Depletion of macrophages due to clodronate liposome administration impaired the repair of fibrotic tissues after liver IRI. In a CCl_4_-induced acute liver injury model, HSCs orchestrated the clearance of damaged tissues by polarizing the macrophage phenotype and facilitated liver remodeling after liver injury (Mochizuki et al., 2014). These findings suggest that HSCs play a role in liver repair after liver IRI through their crosstalk with macrophages (Figure 3).

Analyses by single-cell-RNAseq and spatial mapping have identified HSC zonation across the healthy mouse liver (Dobie et al., 2019; Rosenthal et al., 2021). HSCs include two subpopulations: peri-portal and peri-central HSCs. Peri-portal HSCs express nerve growth factor receptor (Ngfr), while peri-central HSCs express Adamts like 2 (Adamtsl2). The HSCs zonation was conserved in mouse livers treated with CCl_4_, which is characterized by the occurrence of localized injury in the peri-central area. During CCl_4_-induced acute and chronic liver injury, peri-central activated HSCs upregulate pro-fibrogenic genes, including *Col1a1, Col1a2, Col3a1,* and *Acta2*, and produce collagen, and peri-portal activated HSCs upregulate genes related to proliferation, including *Mki67*, and increase their proliferative capability (Dobie et al., 2019). However, more research is required to elucidate the significance of HSC zonation in healthy and acutely stressed livers.

In a murine chronic liver disease model accompanied by the occurrence of tumor growth, two subpopulations of activated HSCs were identified: weakly activated HSCs enriched in genes and pathways related to cytokines and growth factors, and highly activated HSCs enriched in genes related to extracellular matrix-related molecules. Dynamic shifts in activated HSC subpopulations have been demonstrated during the progression of liver fibrosis (Filliol et al., 2022). After partial hepatectomy in mice, HSCs in the remnant liver were identified as cells expressing collagen genes, including collagen α1I) and proliferation, but not classic activated myofibroblasts in liver fibrosis. Notably, the depletion of collagen gene-expressing cells resulted in a delay in liver regeneration after partial hepatectomy, suggesting that hepatic cells expressing collagen genes contribute to liver regeneration. (Kimura et al., 2023). Upon APAP-induced zonal damage in HSCs, peri-central HSCs produced matrix components to repair damaged tissues and restore zonation, and peri-portal HSCs proliferated to produce new HSCs (Ben-Moshe et al., 2022). The heterogeneity of HSCs and their roles during the development of liver IRI and liver repair warrants further investigation.

## Discussion

Following liver IRI, the liver displays a remarkable regenerative ability and can restore its function through the proliferation of hepatocytes, modulation of the inflammatory response, and reconstruction of damaged sinusoids. The capacity of liver restoration from acute liver injury is a critical factor in the recovery from liver IRI. Severe IRI-mediated liver damage induces dynamic changes in subsets, populations, and functions of resident macrophages, LSECs, and HSCs in addition to the recruitment of immune cells. As the process of liver repair involves non-parenchymal cells accompanied by recruited immune cells, successful liver repair after liver IRI requires the coordinated interplay and synergic actions between hepatic resident and recruited cell components.

Recent technological advances, including single-cell and spatial transcriptomic studies, have identified heterogeneity, characterization of spatial distribution, and transcriptional and phenotypic changes in macrophages, LSECs, and HSCs in both steady and diseased livers. Additionally, these new insights into the heterogeneity of liver cell composition during acute liver injury are provided by murine experimental models that underwent partial hepatectomy and were administered with chemicals. However, in the context of liver IRI, it remains to be clarified whether these cell components serve pro-reparative functions to facilitate liver repair and regeneration in both mice and humans. It should also be appreciated that the severity of liver IRI varied from those in partial liver resection or chemical-induced liver injury. Post-IRI livers have large amounts of dead tissue that must be cleared and remodeled. This suggests that the inflammatory and reparative responses of liver-resident and recruited cells to liver IRI would be different, and unrecognized cell subsets that have different functions in liver IRI would be identified by further technological advances. Although the translation of these findings into disease-modifying treatments has proven challenging, a better understanding of the roles of liver non-parenchymal cells would lead to the development of even more targeted therapies.

Furthermore, post-IRI livers display uneven necrotic areas and do not have strict zonations, while chemically treated livers display localized necrosis in the peri-central area. Given that liver zonation is essential for liver homeostasis, the injury and repair of liver zonation during liver IRI warrants further investigation. A deeper understanding of the molecular mechanisms regulating liver zonation would provide therapeutic targets for the promotion of liver repair after liver IRI (Paris and Henderson, 2022).

Although the exact mechanisms underlying liver repair and regeneration is complicated and still not well defined, the advancement of genetic technologies will illustrate comprehensive mechanisms to boost liver repair following hepatectomy and to improve therapeutic options for acute liver failure.
